# Alcohol and Glutamate

**Published:** 1997

**Authors:** Rueben A. Gonzales, Jason N. Jaworski

**Affiliations:** Rueben A. Gonzales, Ph.D., is an associate professor and Jason N. Jaworski is a graduate research assistant in the Department of Pharmacology, College of Pharmacy, University of Texas, Austin, Texas

**Keywords:** glutamate receptors, NMDA receptors, neurotransmission, cell signaling, membrane channel, brain function, neuron, hereditary factors, environmental factors, AODE (alcohol and other drug effects), toxic drug effect, fetal alcohol syndrome, AOD withdrawal syndrome, cognitive process, learning, literature review

## Abstract

Excitatory neurotransmitters, the most important of which is glutamate, increase the activity of signal-receiving neurons and play a major role in controlling brain function. Glutamate exerts its effects on cells in part through three types of receptors that, when activated, allow the flow of positively charged ions into the cell. Of these, the *N*-methyl-d-aspartate (NMDA) receptor plays a particularly important role in controlling the brain’s ability to adapt to environmental and genetic influences. Even low alcohol concentrations can inhibit the excitatory activity of the NMDA receptor. This inhibition of NMDA receptor function may be one of the mechanisms contributing to fetal alcohol syndrome and other more subtle developmental abnormalities. Moreover, alcohol-induced impairment of the NMDA receptor may contribute to alcohol-related learning disabilities, neuronal losses, and cognitive deficits as well as to some of the manifestations of alcohol withdrawal.

Nerve cells, or neurons, transmit signals from the environment to the central nervous system (CNS), among different regions of the CNS, and from the CNS back to other organs (i.e., the periphery). This signal transmission is mediated primarily by small molecules called neurotransmitters. In general, neurotransmitters can be classified as either excitatory or inhibitory. Excitatory neurotransmitters increase and inhibitory neurotransmitters decrease the activity (e.g., the firing rate) of the signal-receiving (i.e., postsynaptic) neuron. Neurons differ in their abilities to recognize, integrate, and pass on the signals conveyed by neurotransmitters. For example, some neurons continually fire at a certain rate and thus can be either excited or inhibited in response to environmental changes. Other neurons normally are at rest. Accordingly, any modification of their activity must occur in the form of excitation. As a result, neuronal excitation plays a fundamental role in controlling brain functioning, and substances that interfere with excitatory neurotransmitter systems can dramatically affect brain function and behavior. Alcohol is such a substance; it appears to interfere with the signal transmission mediated by the neurotransmitter glutamate.

Of the numerous molecules governing normal brain functioning, glutamate (also called glutamic acid) is one of the most important, and research on its functions has generated exciting advances in understanding how the brain works. This article briefly reviews glutamate’s role in normal brain functioning and relates recent findings on how alcohol affects glutamate-mediated (i.e., glutamatergic) signal transmission.

## Glutamate: An Amino Acid Acting as an Excitatory Neurotransmitter

Glutamate’s role as an important signaling molecule has been recognized only within the past two decades. Glutamate is an amino acid; molecules of this type are the building blocks of proteins. Because all cells in the body, including neurons, produce proteins, glutamate is present throughout the brain in relatively high concentrations. Consequently, researchers initially considered glutamate primarily an intermediate metabolic product of many cellular reactions unrelated to neuronal signal transmission and thus did not interpret its presence in neurons as evidence of a potential role as a neurotransmitter.

Although the first indications of glutamate’s excitatory function in the brain emerged in the 1950’s, these findings initially were dismissed: Glutamate application to neurons elicited excitatory responses in virtually every brain area examined, suggesting that this excitation was not a specific response (Collingridge and Lester 1989). Only later did scientists recognize that the observed glutamate effect was indeed valid because excitatory signaling mechanisms had to be operative throughout the CNS. In the 1970’s and 1980’s, researchers identified specific glutamate receptors—proteins on the surface of neurons that specifically bind glutamate secreted by other neurons and thereby initiate the events that lead to the excitation of the postsynaptic neuron. The identification of these glutamate receptors underscored glutamate’s importance as an excitatory neurotransmitter. In addition to glutamate, other structurally related amino acids may have similar excitatory effects on neuronal activity in certain brain regions (Griffiths 1990). However, controversy exists regarding the role of these other excitatory amino acids.

### Glutamate Synthesis, Release, Uptake, and Metabolism

**Figure 1:**
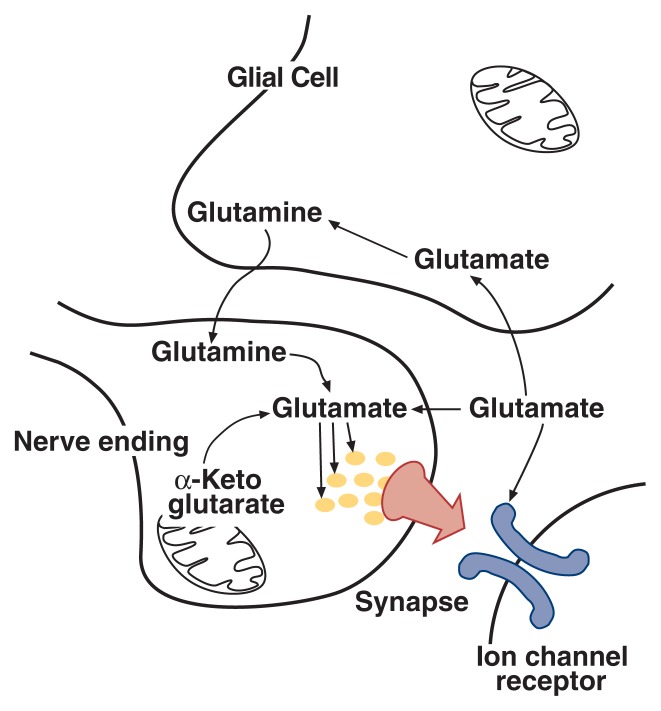
The glutamatergic synapse. The life cycle of the excitatory neurotransmitter glutamate is shown schematically. The precursor molecules a-ketoglutarate and glutamine are converted to glutamate within the nerve ending. The glutamate is stored in synaptic vesicles and delivered to the synapse upon appropriate stimulation. Once in the synapse, glutamate interacts with ion channel receptors to excite the signal-receiving neuron. Glial cells absorb glutamate from the synapse and convert it to glutamine, which may be recycled back to the neuron.

Glutamate is one of the so-called nonessential amino acids^1^—that is, the cells can generate (i.e., synthesize) glutamate from other molecules, primarily α-ketoglutarate and glutamine (Nicholls 1994) (figure 1). The molecule α-ketoglutarate, which can be converted into glutamate in a one-step reaction, is a component of an important energy-producing cycle that occurs in the mitochondria, the cell’s “energy factories.” In contrast, glutamine is provided mostly by cells called glial cells,^2^ which surround neurons. Within the neurons, glutamine also is converted to glutamate in a one-step reaction. The chemical reactions and enzymes involved in glutamate synthesis are not specific to neurons but occur in all cells.

In contrast to other cells in the body, neurons need glutamate not only for normal metabolic activities but also for signal transmission. Accordingly, the glutamate molecules that are destined to act as neurotransmitters must be segregated from the non-neurotransmitter glutamate. To achieve this separation, the neurotransmitter pool of glutamate is stored in special small compartments within the neurons (i.e., synaptic vesicles). Similar synaptic vesicles exist for other neurotransmitter systems. Synaptic vesicles are located in the nerve endings (i.e., the terminal region). When a glutamatergic neuron is activated to pass a signal on to neighboring neurons, the glutamate-containing synaptic vesicles fuse with the membrane of the terminal region and release their contents into the space separating the neurons from each other (i.e., the synaptic cleft). Glutamate then crosses the synaptic cleft to interact with receptors on the postsynaptic neuron, thereby inducing excitation of the postsynaptic neuron.

To terminate this signaling reaction, specific carrier proteins transport glutamate back into the signal-emitting (i.e., presynaptic) neuron and into surrounding glial cells (Nicholls 1994). In addition to preventing excessive excitation of the postsynaptic neuron, this glutamate-uptake system recycles the neurotransmitter pool of glutamate for further use. Glutamate molecules taken back up into the presynaptic neuron are transported directly to the synaptic vesicles. The glutamate transported into glial cells is rapidly converted to glutamine, which eventually can be transported back to the neuron to act as a precursor for glutamate. Thus, glial cells associated with glutamatergic neurons help regulate the signaling process by terminating the synaptic signal and by ensuring a sufficient supply of precursor molecules. This role of glial cells is unique to glutamatergic excitatory neurotransmission; other neurotransmitter systems apparently do not require this type of support from glial cells.

### Glutamate Receptors

In addition to glutamate, the glutamate receptors are the primary molecules involved in excitatory signal transmission (Nakanishi 1992). Glutamate receptors are located on the surfaces of most neurons as well as on some glial cells. These receptors detect glutamate release from neighboring cells (i.e., serve as recognition devices) as well as convert the excitatory signal and relay it into the postsynaptic cell’s interior (i.e., act as signal transducers).

Two major classes of glutamate receptors exist, ion-channel receptors and second-messenger–linked receptors, based on the mechanism used to relay the neurotransmitter signal. Ion-channel receptors respond to glutamate binding by allowing positively charged molecules and atoms (i.e., ions) to enter the cells. This type of glutamate receptor transmits most of the fast-traveling excitatory signals in the CNS. Second-messenger–linked receptors primarily play a regulatory or modulatory role by altering or integrating other signals that the cell receives. When activated by glutamate binding, these receptors cause chemical changes in the cell. Alcohol appears to interfere with the ion-channel type of glutamate receptor, thereby altering primary excitatory signaling throughout the brain. This finding may explain alcohol’s widespread effects on neuronal activity and brain function.

**Figure 2:**
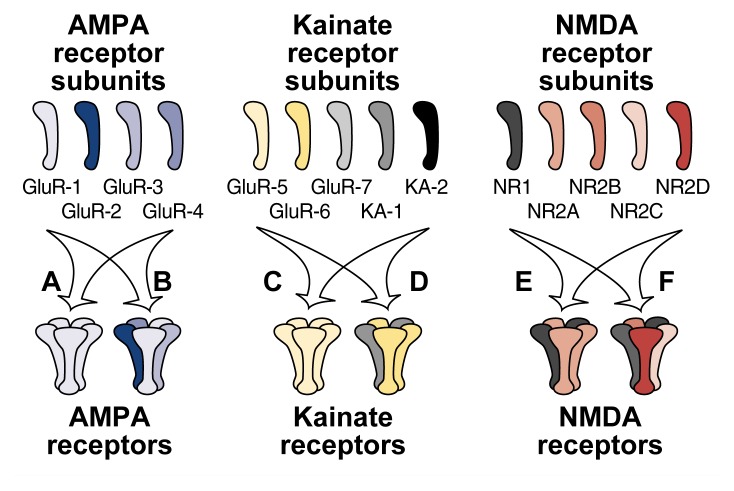
Schematic representation of the hypothetical structure of the three families of glutamate receptors — AMPA, kainate, and NMDA — and their subunits. Each receptor is composed of several subunits that combine to form a channel through which ions can enter the neuron. The different subunits within each family are more closely related to each other than to subunits of other families, and subunits from different receptor families do not combine. AMPA and kainate receptors can consist either of several copies of one subunit (i.e., form homomers) (A and C) or of several different subunits (i.e., form heteromers) (B and D). NMDA receptors appear to form only heteromers and to require at least one NR1 and one NR2 subunit to be functional. Glutamate receptors such as the ones shown here are functional in experimental situations. The precise structure and subunit composition of glutamate receptors in the brain, however, have not yet been determined.

Ion-channel glutamate receptors consist of several closely related proteins (i.e., subunits) that combine to form functional receptor molecules. Three broad categories, or families, of these ion-channel glutamate receptors exist, each of which differ in their subunit compositions (figure 2). Researchers can discriminate among these families with the help of certain compounds that each bind to only one type of receptor. These compounds (and the receptors named after them) are **α**-amino-3-hydroxy-5-methyl-ioxyzole-4-propionic acid (AMPA), kainate, and *N*-methyl-d-aspartate (NMDA). The protein subunits comprising the AMPA and kainate receptors are more closely related to each other than to the subunits making up the NMDA receptors.^3^

When activated by glutamate binding, all three receptor families cause excitation in the postsynaptic cell by allowing positively charged ions (e.g., sodium [Na^+^] or calcium [Ca^2+^]) to enter the cell. This rapid movement of positive ions into the cell reduces the voltage difference that normally exists between the cell’s interior and exterior (i.e., across the cell membrane) adjacent to the receptors. This reduction in voltage difference is called depolarization. Because each neuron carries thousands of glutamate receptors, the ion flow caused by an excitatory signal can result in a depolarization sufficient to generate another excitatory signal in the postsynaptic cell. This second signal is then transmitted to other neurons with which the postsynaptic cell is connected.

Both the AMPA/kainate and NMDA receptors act through the general mechanism of neuronal excitation described above. However, key differences in the properties of these ion channels exist that give rise to specific functional characteristics. For example, most AMPA/kainate receptors allow Na^+^, but little Ca^2+^, into the cells. Moreover, these receptors only require the presence of glutamate to be activated. NMDA receptors, in contrast, allow Na^+^ as well as Ca^2+^ into the neuron and require both glutamate and an additional depolarizing stimulus (e.g., from a previously activated AMPA/kainate receptor) for their activation. The physiological consequences of these differences are that AMPA/kainate receptors are more suited for relaying fast “on/off” signals, whereas NMDA receptors are better suited for functions that require the integration of several converging signals. Consequently, NMDA receptors act as “coincidence detectors” that are activated only when two events (i.e., depolarization and glutamate binding) coincide. This feature makes the NMDA family of glutamate receptors a prime candidate for acting as molecular switches that contribute to many forms of brain “plasticity,” such as learning and memory formation. Conversely, the fast AMPA/kainate receptors likely represent the brain’s primary mediators of excitatory signals.

## The Double-Edged Sword: Physiology and Pathophysiology

The term “plasticity” refers to the brain’s ability to adapt to various environmental or genetic influences. Several types of plasticity exist, including developmental plasticity, long-term potentiation (LTP), and synaptic plasticity in response to injuries. Developmental plasticity occurs during embryonic brain development, when neuronal cells migrate to particular areas of the brain and form innumerable synaptic connections. As the brain’s development progresses, neurons that have formed stable connections with other cells continue to live, whereas neurons that are no longer needed die. Eventually, a stable network of neurons emerges: the normal, functioning brain. NMDA receptors play an important role in these critical developmental processes, and disruption of NMDA receptor function during development can lead to severe and potentially permanent brain dysfunction (Leslie and Weaver 1993).

LTP is a process by which neurons form particularly stable synaptic connections after being repeatedly exposed to specific patterns of stimulation. The concept of LTP can be illustrated with the following, highly simplified example: When learning to ride a bicycle, a child frequently falls because he or she does not know how to maintain balance by shifting his or her weight. The sensory information obtained during these first attempts converges on brain regions involved in controlling movements (e.g., the cerebellum and the cortex). After repeated attempts, stable synaptic connections form between neurons in these brain regions, allowing the rider to automatically shift his or her weight to avoid a fall. Moreover, these synapses are so stable that a person easily can resume riding a bicycle even after many years without practice.

Researchers first observed LTP in certain cells of the hippocampus, a brain region involved in memory formation in humans (Bliss and Collingridge 1993). Accordingly, LTP is the most likely mechanism underlying associative memory formation, which plays an important role in higher forms of learning and cognitive function. However, although LTP has been observed in experiments and possesses many properties corresponding to those involved in memory formation in humans, researchers do not know whether LTP actually occurs during normal, everyday learning. NMDA receptors contribute significantly to LTP, and NMDA receptor-dependent LTP is the most studied and understood form of this phenomenon. However, LTP also may involve receptors for other excitatory neurotransmitters (Bliss and Collingridge 1993).

Synaptic plasticity also can occur in response to neuronal injury. Thus, when neurons are fatally injured, surrounding neurons may rearrange their synaptic connections, presumably to compensate for the loss of a set of synapses. Again, NMDA receptors are known to be involved in this process in response to brain trauma (Schallert and Jones 1993).

These examples underscore the need for adequate glutamatergic neurotransmission to maintain normal physiological brain functioning. However, excessive glutamatergic activity in the brain also can result in serious problems, including the death of neurons. For example, the overstimulation of glutamatergic neurons can lead to seizures. In extreme cases of excessive glutamate receptor activity, direct lethal damage to the neurons (i.e., excitotoxicity) can occur, in which the neurons literally are excited to death. Excitotoxicity may contribute to many types of neurodegenerative disorders, such as Huntington’s or Alzheimer’s disease, as well as to some of the delayed damage caused by brain trauma or strokes (Choi 1992).

In summary, glutamatergic neurotransmission plays a pivotal role in brain function throughout the life span. Glutamate relays excitatory signals between neurons that form specific circuits and whose activation leads to sensations, thoughts, and actions. Glutamatergic neurotransmission also occurs in synaptic connections that mediate the brain’s responses to various environmental and developmental influences. Throughout life, some forms of learning and memory, as well as of higher cognitive functions, likely depend on normal glutamatergic activity. In addition, the brain’s compensatory responses to certain injuries probably are mediated by glutamate-dependent signaling. However, to prevent the overstimulation of neurons, excitatory neurotransmission is tightly regulated. The loss of these regulatory influences may result in pathological conditions, such as seizures or neurodegenerative diseases.

## Alcohol’s Effects on Glutamatergic Signaling

The investigations of alcohol’s potential interactions with glutamatergic signaling have made rapid progress since the late 1980’s, when pharmacological tools became available to distinguish between signals carried through AMPA/-kainate and NMDA receptors. In 1989 the first study was published demonstrating that in rat neurons grown in tissue culture (i.e., in vitro), alcohol concentrations as low as approximately 0.03 percent^4^ directly inhibited ion flow through the NMDA receptor (Lovinger et al. 1989). These findings were particularly significant because they indicated that alcohol concentrations commonly achieved during human alcohol consumption had an inhibitory effect on the NMDA receptor. Under certain conditions, alcohol also may inhibit the AMPA/kainate receptors. For example, in experiments using frog eggs, alcohol inhibited the ion flow induced by low kainate levels to a greater extent than it inhibited the ion flow induced by high kainate levels (Dildy-Mayfield and Harris 1992). These findings suggest that different subtypes of kainate receptors exist with various sensitivities to alcohol. The implications of these finding are unclear, however, because most studies suggest that NMDA receptors are inhibited at lower alcohol concentrations than are non-NMDA receptors.

Researchers have used biochemical as well as electro-physiological techniques to analyze alcohol’s effects on glutamatergic signal transmission in more detail. Biochemical studies generally have demonstrated that alcohol inhibits NMDA receptor function. For example, alcohol decreased the NMDA-induced Ca^2+^ flow into neurons (Leslie and Weaver 1993; Hoffman et al. 1989). Alcohol also interferes with the neurotransmitter release from the postsynaptic neuron that is induced by NMDA receptor activation. Thus, several studies using isolated brain slices found that alcohol reduced the NMDA-induced release of several neurotransmitters, including dopamine, norepinephrine, and acetylcholine (Göthert and Fink 1989; Woodward and Gonzales 1990).

Electrophysiological analyses (i.e., studies measuring the electrical activity of neurons) have supported the biochemical findings. These analyses found that in isolated neurons, neurons within brain slices, and neurons within intact brains (i.e., in vivo), alcohol reduced the excitatory electrical signals evoked by NMDA (Leslie and Weaver 1993; Tsai et al. 1995). The decreased electrical activity may help explain the reduced neurotransmitter release in response to NMDA. This convergence between the biochemical and electrophysiological evidence considerably strengthens the hypothesis that impaired glutamatergic neurotransmission represents a primary molecular mechanism underlying alcohol’s actions on the brain.

Electrophysiological studies also have revealed that not all neurons exhibit altered responses to NMDA after alcohol exposure (Crews et al. 1996). One explanation for this observation is that NMDA receptors differ among neurons. As mentioned earlier, each NMDA receptor consists of several subunits. Five different subunits—NR1 and NR2A to NR2D—exist, which are encoded by five separate genes. Consequently, NMDA receptors on various neurons differ in their subunit compositions. For example, the NR1 subunit is evenly distributed throughout the brain (i.e., is present in each NMDA receptor), whereas the distribution of the different NR2 subunits varies among brain regions and throughout development (Monyer et al. 1994). The exact subunit composition of NMDA receptors is unknown, but functional receptors appear to contain at least one NR1 subunit along with one or more of the NR2 subunits (Monyer et al. 1992). Recent studies have analyzed alcohol’s actions on the NMDA receptor using ifenprodil, an agent that selectively inhibits NR2B subunits (i.e., an NR2B antagonist). These studies found that alcohol specifically inhibited NMDA receptors that were ifenprodil sensitive, whereas receptors insensitive to ifenprodil also were insensitive to alcohol (for a review, see Crews et al. 1996). These findings suggest that at least one NR2B subunit is required for alcohol to affect an NMDA receptor.

Since researchers have proven alcohol’s inhibitory effect on glutamatergic neurotransmission through the NMDA receptors, many studies have tried to elucidate the molecular mechanisms underlying alcohol’s actions. To date, however, these analyses have not yielded unequivocal results (Peoples et al. 1996). Some researchers have used molecular biological techniques to identify specific subunit combinations or regions of the NMDA receptor protein that contribute to the alcohol-induced changes in receptor function. Although certain subunit combinations appear to be more sensitive to alcohol’s inhibitory effects than others, the relevance of these findings will not be clear until the subunits in the intact brain have been clearly defined (Chu et al. 1995). Moreover, the mechanisms underlying alcohol’s effects may differ, depending on the experimental conditions used during these investigations.

## Consequences of Alcohol’s Effects on Glutamate Systems

Intensive research in the past 8 years has suggested that the alcohol-induced inhibition of NMDA receptors underlies many of the pharmacological and toxicological consequences of acute and chronic alcohol consumption. Alcohol may affect several NMDA-mediated processes, including plasticity and excitotoxicity. In addition, inhibition of NMDA receptor function may contribute to some of alcohol’s behavioral effects.

### Effects on Plasticity

The most severe manifestation of alcohol’s effects on developmental plasticity is fetal alcohol syndrome, which is characterized by certain facial abnormalities, growth retardation, and mental retardation. In addition, alcohol-induced inhibition of NMDA receptors during the prenatal period may cause more subtle developmental alterations, such as impairments in learning and memory. Studies in rats have suggested that chronic maternal alcohol consumption causing peak maternal blood alcohol levels as low as 0.04 percent during the last third of gestation^5^ reduces NMDA function in the offspring (Savage et al. 1991). NMDA-dependent plasticity also is reduced in rats prenatally exposed to chronic alcohol (Swartzwelder et al. 1988; Morrisett et al. 1989).

Glutamate function in unborn animals also can be affected by one-time (i.e., acute) alcohol exposure. In pregnant guinea pigs, acute oral alcohol administration resulting in blood alcohol levels of approximately 0.3 percent in both the mother and the fetus produced long-lasting decreases in glutamate release in the fetus but not in the adult (Reynolds and Brien 1994). These findings demonstrate that fetuses may be more sensitive than adults to acute alcohol exposure.

Other studies also have suggested that the consequences of chronic alcohol exposure on NMDA receptors and their functions differ between adults and developing organisms. For example, chronic alcohol-induced inhibition of NMDA receptors during embryonic development may disrupt the normal developmental changes occurring in the NMDA receptor system, eventually resulting in decreased NMDA function in the adult (Savage et al. 1991). Conversely, chronic alcohol consumption in adults may cause an increase in NMDA receptor numbers, possibly to compensate for the continuous alcohol-induced inhibition of receptor function. Thus, the same primary alcohol effect—inhibition of NMDA receptor function—may have different consequences.

Researchers also found that alcohol inhibited NMDA-mediated LTP both in vitro and in vivo. In studies using brain slices isolated from the hippocampus of rats, alcohol prevented LTP, apparently by inhibiting the NMDA receptor-mediated ion flow (Morrisett and Swartzwelder 1993). Similarly, low alcohol doses prevented the development of LTP induced by electrodes implanted into the hippocampus of live rats. Because LTP represents a good model for brain plasticity and may be involved in learning, the alcohol-induced inhibition of LTP may be a mechanism by which alcohol disrupts learning.

### Effects on NMDA-Mediated Excitotoxicity

Some of the deleterious effects of chronic alcohol consumption may result from alcohol’s effects on NMDA-mediated excitotoxicity. Consistent with the finding that alcohol inhibits NMDA receptor function, acute alcohol exposure was shown to reduce excitotoxicity in neurons from the brain’s outer layer (i.e., the cortex) (Tsai et al. 1995). To compensate for chronic alcohol-induced NMDA receptor inhibition, however, the number of NMDA receptors on the cells and, thus, the level of receptor activity increase after long-term alcohol exposure (Grant et al. 1990; Trujillo and Akil 1995). Accordingly, molecular biological studies have demonstrated that chronic alcohol exposure elevates both the mRNA^6^ and protein levels of certain NMDA receptor subunits (Follesa and Ticku 1995; Trevisan et al. 1994; Snell et al. 1996). When alcohol is withheld, however, the NMDA receptors are disinhibited and NMDA receptor activity increases beyond normal levels. This excessive NMDA receptor activity may contribute to seizures and render the cells more susceptible to excitotoxic cell death and thereby may account for some of the manifestations of alcohol withdrawal, such as hyperactivity and seizures. This hypothesis is supported by findings that NMDA antagonists attenuate the severity of alcohol withdrawal seizures. Increased excitotoxic cell death also offers a plausible mechanism for some of the neuronal losses and cognitive deficits associated with chronic alcoholism (Tsai et al. 1995).

### Behavioral Consequences of Chronic Alcohol Consumption

The behavioral relevance of alcohol-induced inhibition of NMDA receptor function has not yet been established. However, intriguing data from some experiments, such as discriminative stimulus experiments, suggest that glutamatergic neurotransmission causes some of alcohol’s behavioral effects. In these experiments, laboratory animals are trained to discriminate between alcohol and water (i.e., to press one lever when given water and a different lever when given alcohol). The animals are rewarded when they press the correct lever. Researchers then can administer various agents (e.g., NMDA receptor antagonists) and determine if the animals perceive these drugs as being similar to alcohol or water. In studies using pigeons, mice, rats, or monkeys, animals that received an NMDA receptor antagonist pushed the alcohol lever a high percentage of the time. These findings suggest that the animals perceived the effects of NMDA receptor antagonists to be similar to those of alcohol (Grant 1994). Given what is known about both glutamate’s role in cognitive processes and the cognitive disturbances caused by acute and chronic alcohol administration, alcohol-induced inhibition of NMDA receptors likely contributes to the cognitive dysfunction associated with intoxication. This hypothesis, however, still requires additional experimental support.

## Conclusions

Over the past decade, scientists have made enormous progress in understanding the role of excitatory amino acids, particularly glutamate, in brain functioning. These scientific advances also have led to a better understanding of alcohol’s effects on excitatory signaling in the brain. The discovery that alcohol inhibits NMDA receptor function has stimulated a stream of research activities. Growing evidence implicates alcohol-induced disruptions of NMDA receptor activity in many of the well-known problems caused by chronic alcohol consumption, including fetal alcohol syndrome, developmental abnormalities, physical dependence, and cognitive disruptions. Future studies will provide additional exciting results concerning alcohol’s interactions with the brain’s primary excitatory neurotransmitter system.

## Footnotes

1 Nonessential amino acids do not have to be taken up with the diet because they can be produced in the body.

2 Glial cells, which constitute the majority of cells in the nervous system, act as intermediaries between blood vessels and neurons, providing the neurons with mechanical support, nutrients, and other substances that may enhance neuronal function.

3 Because the AMPA and kainate receptors are very similar to each other, they will be considered as one group of glutamate receptors for the remainder of this article.

4 In most States in the United States, blood alcohol levels of 0.1 percent are considered legally to be intoxicating.

5 This timeframe roughly corresponds to the fifth and sixth months of a human pregnancy.

6 mRNA is an intermediate molecule generated during the conversion of the genetic information encoded in the DNA into a protein product.
